# A national survey of ambulance paramedics on the identification of patients with end of life care needs

**DOI:** 10.29045/14784726.2020.12.5.3.8

**Published:** 2020-12-01

**Authors:** Peter Eaton-Williams, Jack Barrett, Craig Mortimer, Julia Williams

**Affiliations:** South East Coast Ambulance Service NHS Foundation Trust: ORCID iD: https://orcid.org/0000-0001-5664-3329; South East Coast Ambulance Service NHS Foundation Trust: ORCID iD: https://orcid.org/0000-0002-0040-537X; South East Coast Ambulance Service NHS Foundation Trust: ORCID iD: https://orcid.org/0000-0001-6989-2244; South East Coast Ambulance Service NHS Foundation Trust; Paramedic Clinical Research Unit (ParaCRU), University of Hertfordshire: ORCID iD: https://orcid.org/0000-0003-0796-5465

**Keywords:** continuing professional development, end of life care, palliative care, paramedic, survey

## Abstract

**Objectives:**

Developing the proactive identification of patients with end of life care (EoLC) needs within ambulance paramedic clinical practice may improve access to care for patients not benefitting from EoLC services at present. To inform development of this role, this study aims to assess whether ambulance paramedics currently identify EoLC patients, are aware of identification guidance and believe this role is appropriate for their practice.

**Methods:**

Between 4 November 2019 and 5 January 2020, registered paramedics from nine English NHS ambulance service trusts were invited to complete an online questionnaire. The questionnaire initially explored current practice and awareness, employing multiple-choice questions. The Gold Standards Framework Proactive Identification Guidance (GSF PIG) was then presented as an example of EoLC assessment guidance, and further questions, permitting free-text responses, explored attitudes towards performing this role.

**Results:**

1643 questionnaires were analysed. Most participants (79.9%; n = 1313) perceived that they attended a patient who was unrecognised as within the last year of life on at least a monthly basis. Despite 72.0% (n = 1183) of paramedics indicating that they had previously made an EoLC referral to a General Practitioner, only 30.5% (n = 501) were familiar with the GSF PIG and of those only 25.9% (n = 130) had received training in its use. Participants overwhelmingly believed that they could (94.4%; n = 1551) and should (97.0%; n = 1594) perform this role, yet current barriers were identified as the inaccessibility of a patient’s medical records, inadequate EoLC education and communication difficulties. Consequently, facilitators to performing this role were identified as the provision of training in EoLC assessment guidance and establishing accessible, responsive EoLC referral pathways.

**Conclusions:**

Provision of EoLC assessment training and dedicated EoLC referral pathways should facilitate ambulance paramedics’ roles in the timely recognition of EoLC patients, potentially addressing current inequalities in access to EoLC.

## Introduction

End of life care (EoLC) is a priority for healthcare service providers (Etkind et al., 2017), and is defined as care for adults who are approaching the end of their life, including people who are likely to die within 12 months and people with advanced, progressive, incurable conditions (NICE, 2017). Quality standards for EoLC promote the timely identification of people approaching the end of their lives, subsequent planning for integrated care and an appropriate response to unscheduled support needs at any time of the day and night (NICE, 2017). As a provider and coordinator of emergency and urgent care, the ambulance service is recognised as having a significant role in meeting EoLC standards (National End of Life Care Programme [NEoLCP], 2012). Consequently, a significant amount of ambulance research and service delivery innovation has focused on the management of unscheduled presentations of formally recognised EoLC patients (Kirk et al., 2017; London Ambulance Service NHS Trust, 2019; Munday et al., 2011, cited in Pettifer & Bronnert, 2013; Public Health England, 2014; South Western Ambulance Service, 2017). Yet the role of ambulance services in the initial identification of patients who may be within the last year of their life remains underdeveloped (Long, 2019; NEoLCP, 2012).

Timely identification of EoLC patients is cost effective for the NHS and improves subsequent experiences of healthcare provision for patients (Dixon et al., 2015; Public Health England, 2015). However, many inequalities in access to EoLC services exist (Dixon et al., 2015; Rogers et al., 2019). Frail and elderly patients with multiple comorbidities that do not have a cancer diagnosis are often not identified as being within their last year of life (Public Health England, 2015). Prognostication for this group is difficult, and identification is hindered by a lack of effective communication between healthcare providers (Pocock et al., 2019). There are also inequalities in EoLC access related to geographical, socioeconomic and ethnicity factors (Public Heath England, 2015; Rogers et al., 2019). Ambulance clinicians may become aware of a patient’s requirement for improved care provision, or their increased frequency of unscheduled presentations, at an early stage. Poor physical health status and an increasing number of comorbidities are both associated with increased use of the ambulance service for urgent care needs, and patients from low-income or minority ethnic groups also have an increased likelihood to contact ambulance services over primary care services (Booker et al., 2015). Therefore, improving the appropriate identification and referral of patients with EoLC needs by the ambulance service has the potential to improve recognition rates and address inequalities (Long, 2019; NEoLCP, 2012).

The Gold Standards Framework Proactive Identification Guidance (GSF PIG) (Gold Standards Framework, 2016) is accepted guidance supporting the timely identification of patients within the last year of their life (NICE, 2017), and it is specifically referenced in United Kingdom (UK) ambulance service clinical practice guidelines produced by the Joint Royal Colleges Ambulance Liaison Committee (JRCALC, 2019). The GSF PIG describes general indicators of decline as well as disease-specific indicators for consideration in assessment and there is considerable evidence supporting its effectiveness (Gold Standards Framework, 2016). It is not known to what extent ambulance paramedics currently perform a role in the identification of EoLC patients. Therefore, to inform the development of this practice, this study aims to understand if ambulance paramedics in England come into regular contact with patients who they feel have unaddressed EoLC needs, to learn if they are highlighting this to community healthcare providers and to gauge their awareness of guidance supporting EoLC assessment. Additionally, it aims to explore whether ambulance paramedics believe that a role in identifying EoLC patients is both appropriate and achievable within their clinical practice and to identify any existing barriers and proposed facilitators.

## Methods

A cross-sectional survey of ambulance paramedics was undertaken utilising an online questionnaire. Though surveys are susceptible to poor response rates and bias (Cutter, 2012), this design was chosen to enable a national perspective to be gained. The questionnaire was developed by the research team and pre-tested on five practising paramedics from the same trust to ensure both its clarity of meaning and its appropriateness to evaluate the study’s aims. Repeated promotion of participation, questionnaire conciseness and anonymity was employed to reduce bias (Cutter, 2012). Paramedic registration was chosen as an inclusion criterion because paramedics are most commonly the clinicians responsible for on-scene decision-making and paramedic-led care is advocated for all ambulance service patients (College of Paramedics, 2017). Though non-registered ambulance clinicians also perform patient assessment and onward referral, analysis of the variable of clinician grade was beyond the scope of this study. Paramedics working in other healthcare environments were excluded because this study is focused solely upon ambulance-based clinical practice.

The nine participating English NHS ambulance service trusts promoted the questionnaire using a poster and email invitation supplied by the research team and disseminated through their existing communication channels. The questionnaire was accessible on the GDPR-compliant (HM Government, 2018) survey platform Online Surveys (www.onlinesurveys.ac.uk) from 4 November 2019 until 5 January 2020. Data were collected anonymously, as the name of the participant’s employing trust was the only demographic information requested. Following initial multiple-choice assessment of current practice and awareness, participants were invited to review the GSF PIG (2016) within the survey. Subsequently, further questions, permitting free-text replies, addressed their attitudes towards GSF PIG utilisation within ambulance paramedic practice. All data collected and downloaded were password protected and analysed solely by the research team. Descriptive statistics were employed to analyse multiple-choice responses, and content analysis was applied by the lead author to free-text data in order to enrich exploration of participants’ attitudes (Vaismoradi et al., 2013). Descriptive content analysis quantifies the number of times that a subject or ‘category’ is submitted by participants, and these may then be grouped together within themes (Bengtsson, 2016).

All participants were presented with a participant information sheet (PIS) before commencing the questionnaire, informing them that submitting a completed questionnaire indicated consent and that withdrawal from the study was subsequently unavailable due to the anonymous nature of data collection. Participants were instructed not to disclose any person-identifiable information in free-text responses. Participation was not anticipated to cause distress, but the PIS highlighted employers’ welfare services if required. The study gained NHS Health Research Authority (HRA) approval (IRAS: 268490) and all participating trusts formally agreed participation in accordance with HRA procedure.

## Results

1653 questionnaires were collected and 1643 were analysed; all were completed in their entirety. 10 questionnaires were excluded because personnel from non-participating trusts completed them. The overall response rate, calculated from the registered paramedic population reported as employees by trusts, was 11%. Recruitment strategies varied across trusts and while an element of nonresponse bias may exist, this does not invalidate results (Meterko et al., 2015).

### Current practice

**Figure 1:**
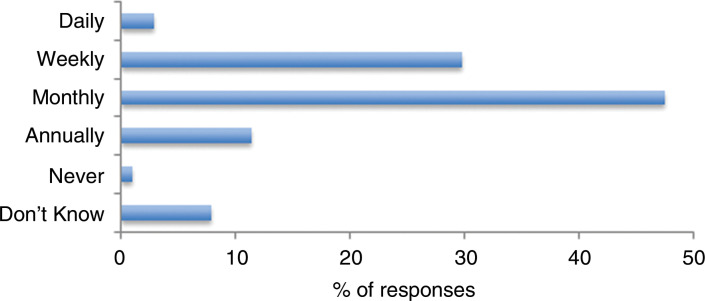
Figure 1. How often do you attend patients who you suspect are in their last year of life who have not been formally recognised as such by the health care system?

Initially, participants were asked to estimate the frequency of their encounters with patients who they believed might be in the last year of life but who were not formally recognised as such by the healthcare system. 79.9% (n = 1313) perceived that this happened on at least a monthly basis. Only 1.0% (n = 16) replied that they did not encounter such patients, with a further 7.8% (n = 128) indicating that they did not know (Figure 1).

**Figure 2:**
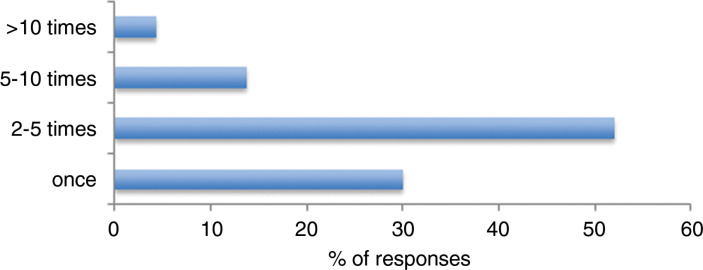
Figure 2. If you have referred a patient to their GP, specifically for the purposes of assessing EoLC needs, how many times have done this in the last 12 months?

72.0% (n = 1183) of participants indicated that they had previously referred a patient to their General Practitioner (GP) specifically for the purpose of EoLC needs assessment. 82.0% (n = 970) of those who had estimated that they had done this between one and five times in the last 12 months (Figure 2).

### Current awareness

**Figure 3:**
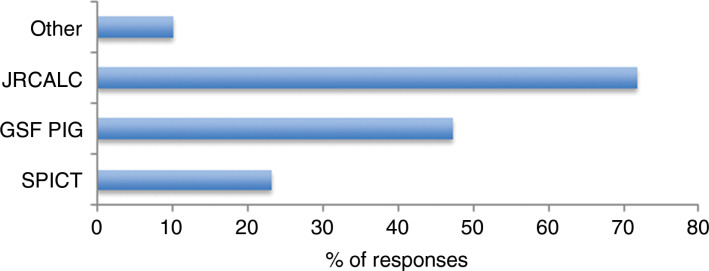
Figure 3. If knowledge of an EoLC assessment guidance informed your decision to refer a patient to their GP, specifically for the purposes of assessing EoLC needs, which guidance are you familiar with?

62.0% (n = 733) of the paramedics who had made an EoLC referral indicated that their decision to do so had not been informed by knowledge of a specific EoLC assessment guidance. Of the 38.0% (n = 450) who reported that knowledge of guidance did support their referral assessment, 71.8% (n = 323) were familiar with guidance within JRCALC UK Ambulance Services Clinical Practice Guidelines (JRCALC, 2019); 47.3% (n = 213) were familiar with the GSF PIG (Gold Standards Framework, 2016); 23.3% (n = 105) were familiar with the Supportive and Palliative Care Indicators Tool (SPICT^TM^, 2020); and 10.2% (n = 46) cited other sources of information and experience (Figure 3).

Of all participants, only 30.5% (n = 501) were aware of the GSF PIG, and of those only 25.9% (n = 130) had received training in its use.

### Current attitudes

Participants were then asked to read a copy of the GSF PIG (Gold Standards Framework, 2016) before continuing with the questionnaire. Following this presentation, 94.4% of participants (n = 1551) indicated that they believed ambulance paramedics can effectively use guidance, such as the GSF PIG, to refer appropriate patients to their GPs for assessment of end of life care needs. From the 92 participants who believed paramedics could not, 86 free-text responses were submitted in explanation. The three most common reasons given were that more training was required (n = 30), the GSF PIG was too complicated (n = 17) and that paramedics had limited access to a patient’s medical records (n = 14).

When asked if ambulance paramedics should contribute to identifying patients who may be in the last year of life for assessment of EoLC needs, 97.0% (n = 1594) of participants felt that they should. 43 free text responses were collected in explanation of why they should not, the most common (n = 25) reason being a perception that this was a community healthcare provider role rather than one suitable for emergency medical services (EMS).

587 free-text responses were submitted by participants in a final ‘further comments’ box. The three themes relating to the most common responses are presented with quotes selected to demonstrate representative content:

1. EoLC clinical education: The most frequent theme (n = 139) was a perceived need for further education of ambulance clinicians in the assessment and management of EoLC patients. This was highlighted for both pre-registration education and trust-facilitated continuing professional development (CPD).
  I think there needs to be more focus in pre-registration training in natural trajectories of disease, frailty, ageing, appropriateness of care (with focus on comfort measures) and MDT working. (Respondent ID: 515487-515478-52279628)
  Consider online bespoke training package, equivalent of level 5 study to give staff the level of understanding required to put this into practice. (Respondent ID: 515487-515478-51942295)
  Some indicated that effective EoLC assessment and management required a shift in the traditional ‘life-saving’ mindset of EMS personnel, resulting in a lack of confidence dealing with this patient group that may diminish with experience (n = 25). A better understanding of trajectories of decline and the likely benefits of intervention were highlighted as requirements not only for clinicians but also for patients and their families and/or carers (n = 23).
2. Ambulance clinical setting: Many paramedics believed that ambulance-based clinical practice provides a unique opportunity to identify appropriate patients for further EoLC assessment (n = 95).
  Paramedics are frequently the only healthcare professionals to see patients in their own home environment and who spend time talking – with permission from the patient – to the friends and relatives of the patient. This puts us in a unique position to truly assess the impact of disease or deterioration on an individual’s daily life. (Respondent ID: 515487-515478-53522978)
  Despite some indicating that the identification of EoLC patients should be a role performed by primary care services (n = 40), there was also a recognition that patients are currently slipping through the net (n = 25) and that appropriate identification will both benefit patient experience and reduce demand on ambulance resources (n = 23).
  We often see people who may slip under the radar of their GP. Every HCP has a role to play in recognising EOL patients and making sure they, and the families and carers, receive adequate support. We need to get away from everyone thinking it’s someone else’s issue to pick up. (Respondent ID: 524372-524363-51622835)
  However, current barriers to performing this role were reported as on-scene time constraints (n = 21), a perceived absence of both pre- and post-decision-making support from employing trusts (n = 35) and limited access to a patient’s medical records (n = 27). Additionally, several respondents suggested that it would be beneficial for the GSF PIG to be simplified and/or adapted specifically for ambulance practice (n = 22).
3. EoLC referral pathways: A significant facilitator to performing this role was identified as a dedicated EoLC referral pathway that was both accessible at all hours and adequately responsive to meet urgent care requirements (n = 97).
  A clear pathway for ambulance crews to speak to a GP both in office hours and out of office hours. Direct pathways to enable district nurses / palliative nurses to be mobilised in the case of no EOL care in place. (Respondent ID: 515487-515478-51964452)
  Some suggested that ambulance trusts themselves could possess dedicated palliative care resources to benefit responsiveness (n = 18), especially related to another category highlighting the absence of available ‘Just in Case’ (JIC) medications (n = 22). A final, unexpected category related to referral was the perception that currently other healthcare professionals often did not recognise paramedics as professionally competent to assess patients as potentially possessing EoLC needs (n = 36).
  GP and primary care services take what paramedics say with a pinch of salt. (Respondent ID: 515487-515478-53530388)

## Discussion

### Practice

The frequency with which paramedics perceive that they encounter patients who they believe are approaching the end of their lives is congruent with earlier surveys exploring EoLC encounters of ambulance clinicians in the West Midlands (Munday et al., 2011, cited in Pettifer & Bronnert, 2013) and the North of England (Kirk et al., 2017), though specifying patients who have not been formally recognised as such within this study prevents direct comparison. The frequency of perceived encounters with these patients together with the free-text theme suggesting that they are in a unique clinical position supports the view that ambulance clinicians can play an important role in improving access to end of life care services (Long, 2019; NEoLCP, 2012). Furthermore, the number of participants who had made EoLC GP referrals and the frequency with which they had done this within the last year provides evidence that identification of EoLC patients is currently being performed within ambulance services in the UK.

### Awareness

Despite paramedics reporting that they are identifying EoLC patients, most participants indicated that they are doing this without knowledge of validated EoLC assessment guidance. As a result, it might be hypothesised that these referrals are occurring towards the later stages of decline when signs of end of life are most apparent. While still of considerable benefit to these patients who are without EoLC provision, it may be that enhanced familiarity with validated guidance is able to improve the timeliness of recognition by ambulance personnel (Gold Standards Framework, 2016).

Paramedics using validated guidance to support referrals were most commonly familiar with the guidelines that have been adapted for ambulance practice contained within the JRCALC Clinical Guidelines (JRCALC, 2019). When considered with responses in free text concerning the complexity and perceived requirement for detailed patient records present within the GSF PIG, this may support further development and promotion of EoLC identification guidance specifically for ambulance clinicians – a recommendation also made by the National End of Life Care Intelligence Network (Public Health England, 2014, p. 8).

Another observation of note is that despite some participants having an awareness of the GSF PIG, only around a quarter of those reported that they had completed training in its use. This equates to just 7.9% of all participants receiving training in application of the GSF PIG. It is possible that some participants may have completed alternative EoLC assessment training. However, when examined in conjunction with the free-text theme of a significant need for more education, this highlights an apparent absence of formal education in the initial identification of patients appropriate to access EoLC services (Hunter & Orlovic, 2018; Kirk et al., 2017; Pettifer & Bronnert, 2013). Provision of such education might also be expected to improve recognition from other healthcare professionals of paramedics’ competence to perform this role.

### Attitudes

Overwhelmingly, paramedics in this study believed that the proactive identification of patients with EoLC needs was both an achievable and appropriate role for their clinical practice. Many of the subject categories identified in this study, related to performing this role, have been discussed previously when considering the wider aspects of EoLC within ambulance-based practice (Kirk et al., 2017; Pentaris & Mehmet, 2019; Pettifer & Bronnert, 2013). Kirk et al. (2017) highlighted poor education for both clinicians and relatives and a perceived lack of organisational support for complex decision-making leading to low confidence in dealing with this patient group, also suggesting this diminished with experience. Pentaris and Mehmet (2019) discussed the need for all healthcare professionals to accept a change in mindset away from the biomedical model and to develop the emotional resilience to accept unavoidable death within their clinical practice. Pettifer and Bronnert (2013) stressed the difficulties of accessing both patient information and responsive specialist services. The latter is one of the key themes emerging from this study as, alongside education, a significant facilitator to performing this role was identified as the provision of dedicated, accessible at all hours and sufficiently responsive referral pathways. The perceived need for responsive access to decision-making support and crisis medication led some to suggest that dedicated palliative resources are provided by ambulance services themselves. This integration of health and care providers remains a priority to facilitate effective EoLC provision (Hunter & Orlovic, 2018).

### Strengths and limitations

Surveys employing volunteer sampling will have an inherent bias towards those with an interest in the subject, and the response rate of this study demands that this is considered (Cutter, 2012). As the dispatch of paramedics is generally performed irrespective of presenting complaint, the frequencies of EoLC patient encounters will likely be representative. However, if our sample represents those with enhanced sensitivity to EoLC issues, frequencies of referral may be less generalisable. Similarly, positive attitudes towards the role of performing EoLC identification may be overrepresented. Yet awareness of assessment guidance may also be overestimated and consequently the requirement for education is more strongly emphasised. Due to the anonymous nature of data collection, the paramedic registration of respondents could not be ensured, though this seems unlikely to have influenced results significantly. Clear anonymity is likely to have strengthened the validity of free-text responses, and the participation of nine ambulance service trusts has enabled a national perspective to be gained.

## Conclusions

Ambulance paramedics frequently encounter patients that they perceive are not receiving appropriate EoLC support, and many are currently referring these patients to their GPs for further assessment of their care requirements. However, most referrals are currently being done without knowledge of validated EoLC assessment guidance. Predominantly, ambulance paramedics believe this is a role both appropriate to and achievable within their clinical environment. Yet the inaccessibility of comprehensive patient records, poor communication channels and a perceived lack of the required responsiveness to EoLC referrals are current barriers to effective performance. Therefore, it is likely that timely identification of EoLC patients within ambulance-based clinical practice would be facilitated by the provision of formal EoLC education and the establishment of dedicated, accessible and responsive referral pathways. Future qualitative and quantitative evaluation of local initiatives providing both assessment training and referral pathways would be hugely beneficial to reveal the benefits and barriers associated with this role in practice.

## Author contributions

PEW was involved in study conception, design, data collection and analysis, together with the drafting and amending of this submission. JB, CM and JW performed critical review of design, analysis and submission. PEW acts as the guarantor for this article.

## Conflict of interest

JW is on the editorial board of the *British Paramedic Journal* but had no input into any decision around this article. JW is also Head of Research for the College of Paramedics, but the funding award was made by anonymous review of proposals by other reviewers.

## Ethics

NHS REC approval was not required; NHS ambulance service trusts formally confirmed participation by completing and returning Organisation Information Documents.

## Funding

This study received funding from the College of Paramedics’ small research grant funding scheme in 2019.
